# Risk factors for transmission of carbapenem-resistant *Acinetobacter baumannii* in outbreak situations: results of a case-control study

**DOI:** 10.1186/s12879-024-09015-7

**Published:** 2024-01-23

**Authors:** Beate Schlosser, Beate Weikert, Giovanni-Battista Fucini, Britta Kohlmorgen, Axel Kola, Anna Weber, Norbert Thoma, Michael Behnke, Frank Schwab, Petra Gastmeier, Christine Geffers, Seven Johannes Sam Aghdassi

**Affiliations:** 1 Institute of Hygiene and Environmental Medicine, Charité– Universitätsmedizin Berlin, corporate member of Freie Universität Berlin and Humboldt- Universität zu Berlin, Hindenburgdamm 27, 12203 Berlin, Germany; 2 BIH Charité Digital Clinician Scientist Program, Berlin Institute of Health at Charité– Universitätsmedizin Berlin, BIH Biomedical Innovation Academy, Anna-Louisa-Karsch-Straße 2, 10178 Berlin, Germany

**Keywords:** Carbapenem-resistance, Acinetobacter Baumanii, Outbreak, Transmission, Risk factor, Infection control

## Abstract

**Background:**

An increase in patients with multidrug-resistant organisms and associated outbreaks during the COVID-19 pandemic have been reported in various settings, including low-endemic settings. Here, we report three distinct carbapenem-resistant *Acinetobacter baumannii* (CRAB) outbreaks in five intensive care units of a university hospital in Berlin, Germany during the COVID-19 pandemic.

**Methods:**

A case-control study was conducted with the objective of identifying risk factors for CRAB acquisition in outbreak situations. Data utilized for the case-control study came from the investigation of three separate CRAB outbreaks during the COVID-19 pandemic (August 2020– March 2021). Cases were defined as outbreak patients with hospital-acquired CRAB. Controls did not have any CRAB positive microbiological findings and were hospitalized at the same ward and for a similar duration as the respective case. Control patients were matched retrospectively in a 2:1 ratio. Parameters routinely collected in the context of outbreak management and data obtained retrospectively specifically for the case-control study were included in the analysis. To analyze risk factors for CRAB acquisition, univariable and multivariable analyses to calculate odds ratios (OR) and 95% confidence intervals (CI) were performed using a conditional logistic regression model.

**Results:**

The outbreaks contained 26 cases with hospital-acquired CRAB in five different intensive care units. Two exposures were identified to be independent risk factors for nosocomial CRAB acquisition by the multivariable regression analysis: Sharing a patient room with a CRAB patient before availability of the microbiological result was associated with a more than tenfold increase in the risk of nosocomial CRAB acquisition (OR: 10.7, CI: 2.3–50.9), while undergoing bronchoscopy increased the risk more than six times (OR: 6.9, CI: 1.3–38.1).

**Conclusions:**

The risk factors identified, sharing a patient room with a CRAB patient and undergoing bronchoscopy, could point to an underperformance of basic infection control measure, particularly hand hygiene compliance and handling of medical devices. Both findings reinforce the need for continued promotion of infection control measures. Given that the outbreaks occurred in the first year of the COVID-19 pandemic, our study serves as a reminder that a heightened focus on airborne precautions should not lead to a neglect of other transmission-based precautions.

## Background

Healthcare-associated infections caused by carbapenem-resistant *Acinetobacter baumannii* (CRAB) are associated with inferior outcomes and increased mortality in affected patients [1]. Given its complex resistance mechanisms and complicated treatment, CRAB is frequently considered as an emerging public health concern [2, 3]. The proportion of CRAB isolates from invasive infections has been demonstrated to be highly variable across European countries, with Germany being among the countries with the lowest proportion [4, 5]. Accordingly, CRAB transmissions occurring in the context of healthcare-associated outbreaks, may account for a substantial share of the overall CRAB burden in Germany. Therefore, and due to the fact that outbreaks with CRAB and other multidrug-resistant organisms (MDRO) frequently occur in particularly vulnerable populations (e.g. immunocompromised patients) [6], understanding the mechanisms of such outbreaks and designing adequate containment measures, is a key objective of infection prevention and control (IPC) activities [5, 7, 8]. The high tenacity of *A. baumannii* on inanimate surfaces renders CRAB a particularly difficult to contain pathogen in healthcare settings and further increases its potential for transmissions and outbreaks [9, 10].

The COVID-19 pandemic necessitated a strong focus of IPC staff and frontline healthcare workers on droplet and airborne transmission precautions. It is conceivable that the heightened focus on preventing the spread of viral respiratory infections could have reduced the awareness of the threat posed by the spread of MDRO in some situations. Furthermore, the increased workload for healthcare workers as well as staff shortages associated with the pandemic probably led to a decrease in adherence to IPC practices [11, 12]. Several MDRO outbreaks in hospitals that have been reported since the onset of the pandemic [13–15], support this notion.

Despite these reports, data on the driving forces of ongoing transmission during CRAB outbreaks, particularly within the context of the COVID-19 pandemic, are scarce. Consequently, the study at hand aims to contribute important data on the matter by investigating risk factors for nosocomial acquisition of CRAB through a matched case-control study conducted as part of the management of three distinct CRAB outbreaks at a university hospital.

## Methods

### Setting

Charité is a university hospital with over 3,000 beds, located at three separate sites in Berlin. Screening for CRAB outside of outbreaks was performed upon admission for patients with selected risk factors (history of MDRO carriage or contact to healthcare facilities outside of Germany) until the end of 2020. In the context of the COVID-19 pandemic, all Charité ICU were advised to screen all admitted patients for MDRO from January 1, 2021 onwards. The institutional IPC guidelines developed by the hospital IPC team detailing recommended IPC measures, were digitally available to all staff at Charité. Additionally, on site IPC training of frontline staff by Charité’s IPC team was intensified during the COVID-19 pandemic.

To facilitate early outbreak detection, Charité’s IPC team employs an automated cluster detection system [16]. Early detection of outbreaks is based on bacterial species and similarities in antimicrobial resistance profiles. Biogenetic sequencing is frequently used to identify underlying transmission events or a common source to confirm outbreaks.

The three independent CRAB outbreaks included in this study involved five intensive care units (ICU) and occurred between August 2020 and March 2021. During the study period, the institutional IPC guidelines in force required enhanced barrier precautions for known or suspected carriers of CRAB. These included isolation in a single room or as part of a cohort, as well as the use of protective gowns and gloves by healthcare workers. Medical devices and other equipment that were exposed to a CRAB patient were to be used strictly for the respective patient and either reprocessed or discarded after use or patient discharge. For devices where such an allocation was not possible (e.g. ultrasound), thorough disinfection after use had to be performed. Frequent environmental cleaning of CRAB patient rooms was recommended. As an additional measure, CRAB patients in ICU were cared for by specifically designated nurses.

### Database

Data utilized in this study originated from two sources. First, various data were collected prospectively for every CRAB outbreak patient as part of the routine outbreak management. These were clinical presentation (colonization vs. infection), age, sex, admission date, time between admission and first detection of CRAB, number and date of CRAB-negative screening swabs, length of stay, death, relevant contact (i.e. sharing of patient room before availability of the microbiological report) to CRAB patients, and treatment in a patient room where a CRAB patient had been treated previously. In addition to the prospectively collected data, selected parameters of interest were collected retrospectively for the purpose of the case-control study (see below).

Outbreak ascertainment was performed at the discretion of the IPC team taking available results of the points indicated above as well as the epidemiological constellation into account. Outbreak containment measures were determined by the IPC team and staff from the affected ICU. Applied mitigation strategies encompassed strict contact precautions for cases and their contacts, weekly screening of all patients for MDRO, taking environmental samples, and intensified cleaning procedures. All three outbreaks were reported to the responsible public health departments.

### Case-control study

A case-control study was conducted with the goal of identifying risk factors for CRAB acquisition in outbreak situations. Cases were defined as patients with hospital-acquired (according to assessment by IPC physician in charge, typically first detection on day two after admission or later) CRAB that were part of one of the three outbreaks. Per outbreak, one patient was determined by the IPC team as the probable index case, and consequently excluded from the case-control study.

The assignment of control patients to cases and the retrospective extraction of parameters was supported by the “Hygieneportal”, an IPC data warehouse developed and maintained by the Institute of Hygiene and Environmental Medicine at the Charité [16, 17].

Per definition, control patients did not have any CRAB positive microbiological findings in their known patient history. To ensure a similar exposure time to the outbreak ward, controls had to be hospitalized on the same outbreak ward during the outbreak period (from admission of the first CRAB outbreak case until discharge of the last outbreak case) for a duration of at least 80% of the time that elapsed between admission and first CRAB detection of the associated case. Control patients were matched retrospectively in a 2:1 ratio (two controls per case).

The parameters collected for cases as part of the outbreak management (see above) were retrospectively collected for controls as well. In addition to these, additional parameters were retrospectively researched for the case-control analyses. For every case and control, it was investigated whether they underwent certain intensive care medical procedures or treatments. These included: prone position, hemodialysis, invasive mechanical ventilation, bronchoscopy, extracorporeal membrane oxygenation (ECMO) and antimicrobial treatment. Additionally, primary diagnosis, Charlson Comorbidity Index [18], and COVID status (i.e. COVID-19 as primary diagnosis) were recorded based on corresponding diagnostic codes during the ICU stay. Moreover, the number of roommates per patient, and the burden of CRAB at the treating ward, defined as the number of patients with CRAB present on the same ward as a case or a control, were determined. The observation period, in which all listed parameters were collected, was defined as follows: For CRAB cases, from outbreak ward admission until the first detection of CRAB, and for controls from outbreak ward admission until discharge from the outbreak ICU.

### Statistical analysis

For continuous parameters, results are shown as median with interquartile range (IQR) and for categorical parameters as number and percentage. For categorical parameters, differences were tested with chi-squared test and for continuous variables with Wilcoxon rank-sum test, respectively. To analyze risk factors for CRAB acquisition, univariable and multivariable analyses to calculate odds ratios (OR) and 95% confidence intervals (CI) were performed using a conditional logistic regression model. In the multivariable analysis, all parameters with a *p* < 0.05 in the univariable model were considered in the multivariable analysis and parameter selection was stepwise forward. Statistical results were considered significant with a *p* < 0.05. All analyses were exploratory in nature and performed using SPSS 26 (IBM SPSS statistics, Somer, NY, USA) and SAS 9.4 (SAS Institute, Cary, NC, USA).

### Ethical statement

The Ethics committee of Charité-Universitätsmedizin Berlin approved the study (EA4/159/21), and data collection was conducted in alignment with the German Protection Against Infection Act (“*Infektionsschutzgesetz*”) and local infection control regulations.

## Results

The three outbreaks took place between August 3, 2020 and March 24, 2021, and contained 26 patients with nosocomial CRAB acquisition from five different ICU. Each outbreak was attributable to a distinct outbreak strain. “Outbreak A” affected 11 patients in a medical ICU at hospital site A with a focus on infectious and pulmonary diseases. While six patients were only colonized, five patients showed signs of an invasive infection with the outbreak strain. “Outbreak B” comprised four patients in two ICU (medical, surgical) at hospital site B, with three patients showing signs of a CRAB infection. “Outbreak C” affected 11 patients in two ICU (surgical, anesthesiological) at hospital site C. Eight patients in outbreak C were infected with the outbreak strain, while three were only colonized. Further key characteristics of the three outbreaks are depicted in Table 1.

**Table 1:** Characteristics of three carbapenem-resistant acinetobacter baumannii (CRAB) outbreaks

Parameter	Outbreak A	Outbreak B	Outbreak C
N and specialty of affected ICU:	1 (Infectious & pulmonary diseases)	2 (Surgery; Cardiology)	2 (Surgery; Anesthesiology)
N of nosocomial cases (N per affected ICU)	11 (11)	4 (1 vs. 3)	11 (4 vs. 7)
N of patients with infection vs. colonization*	5 vs. 6	3 vs. 1	8 vs. 3
First detection of CRAB in			
blood culture	2	0	1
abdomial swab/tissue	0	1	2
rectal swab	2	0	2
pharyngeal swab	1	0	3
perineum/groin swab	0	0	1
respiratory materials	6	3	2
Start date outbreak**	September 22, 2020	August 03, 2020	December 14, 2020
End date outbreak***	January 12, 2021	October 27, 2020	March 24, 2021
Total outbreak duration in days	113	86	100
Patient screening frequency	Admission + twice a week	Admission + weekly	Admission + weekly
N of environmental samples taken (positive for CRAB)	107 (2)	40 (0)	230 (6)
CRAB positive environment samples	mobile storage for continuous hemodialysis filtration device (*n* = 1) ECMO machine before use (*n* = 1)	-	medication storage (*n* = 3) bed side monitor (*n* = 1) emergency cart (*n* = 1) operating button for endotracheal suction (*n* = 1)
Sequence type (ST) of outbreak strain; detected carbapenemase	ST 78; OXA-72	ST 2; OXA-23	ST 2; OXA-23, NDM

* The distinction between infected and colonized patients was based on the assessment of the treating physicians and the infection control team.

**Date of CRAB detection of first nosocomial case.

***Defined as discharge of the last CRAB patient from the outbreak ward.

## Discussion

Our study was able to identify two principal risk factors for nosocomial CRAB acquisition in outbreak situations, sharing a patient room with a CRAB patient, and undergoing bronchoscopy. While the former confirms rather intuitive assumptions, the latter indicates that pathogens in outbreaks can spread in different ways, for instance in the context of using medical devices [13, 19, 20].

Several factors in shared patient rooms likely increased the risk of pathogen transmission, such as the shared use of materials, storage space and surfaces [21–25]. In our study, transmission due to simultaneous admission in patient rooms with CRAB patients likely occurred before CRAB detection, since IPC guidelines at Charité stipulated that all CRAB patients were placed in single rooms with increased barrier precautions. Despite intensive screening, it must be assumed that patients carried and potentially shedded CRAB before the first detection, rendering it difficult to estimate the actual duration of contact that lead to transmission. Despite this challenge, it is important to point out that transmission in shared rooms generally could be prevented by standard IPC precautions, most importantly proper hand hygiene, disinfection of contaminated surfaces and items, as well as appropriate use of personal protective equipment. Several publications indicate that breaches in standard IPC precautions are the most common cause for the spread of pathogens [26, 27]. Poor hand hygiene facilitates transmission of pathogens directly from patient to patient, or indirectly to the inanimate environment [24, 28]. Improving hand hygiene compliance has repeatedly been demonstrated to significantly reduce the spread of pathogens, both within and outside of outbreaks [29, 30]. Similarly, the importance of environmental cleaning and disinfection of surfaces has been demonstrated multiple times to play a key role in preventing the spread of pathogens and in the context of outbreak containment [9, 31]. This is particularly relevant given the high tenacity of *A. baumannii* on inanimate surfaces [32]. We consider this finding of our study to be a stark reminder of the importance of these basic IPC precautions.

From a variety of procedures considered, bronchoscopy was identified as a risk factor for acquiring CRAB. When interpreting this result, it is important to take into account that bronchoscopes used during the outbreak either were single-use devices or reprocessed in the central sterilization unit of the hospital. Given the high degree of validity of reprocessing in the central sterilization unit [33], we believe that deficiencies in the handling of the mechanical equipment required for bronchoscopy may have increased the risk of transmission. The additional medical equipment required for bronchoscopy is usually shared between patients, which could represent a relevant vector for pathogen transmission. Alternately, it is possible that bronchoscopy was mostly a reflection of a higher severity of disease leading to more measures and manipulations performed on the patient and thereby increasing the risk of pathogen transmission. However, the fact that other invasive procedures (e.g. invasive mechanical ventilation, ECMO) were not demonstrated by multivariable analysis to be independent risk factors for CRAB acquisition, and that morbidity measured with the Charlson Comorbidity Index did not differ significantly between cases and controls, does not support this interpretation.

It is remarkable that three separate outbreaks occurred at our hospital during a rather short period in the first year of the COVID-19 pandemic in Germany [34]. There are numerous reports on the occurrence of MDRO outbreaks associated with early phases of the COVID-19 pandemic [13, 35, 36]. Excessive use of antibiotics has been cited as a possible cause [37–39]. Moreover, improper IPC measures by healthcare workers, placing a high focus on airborne transmission routes, but neglecting contact precautions, have also been discussed as potential explanations for this phenomenon [40]. In particular, the risk of spread through contaminated PPE might be underappreciated. The likelihood of PPE as an important vector for MDRO transmission might have been increased during the first year of the COVID-19 pandemic, potentially due to not doffing PPE after individual patients or reusing PPE intended for single use. Moreover, there was a marked lack of staff trained in the oftentimes demanding procedures in ICU (e.g. frequent repositioning of patients, complex invasive interventions, ECMO). Additionally, these procedures likely have led to deviances from the recommended designation of nurses to CRAB patients. It can be assumed that these complicating factors worsened the already high workload in ICU. High workload and inexperienced staff have been demonstrated to be associated with lower IPC compliance and an increased risk of nosocomial infection or pathogen transmission [11, 39, 41]. Overall, however, any link established between the occurrence of the three outbreaks and the COVID-19 pandemic remains speculative.

When interpreting the study results, various limitations must be acknowledged. First, although the number of CRAB patients in our outbreaks was relatively high, it may still be comparatively small for a reliable analysis of risk factors. Second, for controls, data for the case-control study were collected until discharge from the outbreak ward. For cases, data was by definition only collected until the first detection of CRAB, resulting in an overall shorter observation period in cases. Third, some of the data included in the analysis were collected retrospectively, introducing a potential of error due to incomplete documentation. Furthermore, some data were collected prospectively for CRAB cases, but retrospectively for controls, potentially distorting the reliability of information between the two groups. Forth, the considered risk factors represent only a subset of potentially relevant factors and were chosen based on availability in the patient record and the IPC data warehouse. Moreover, the Charlson Comorbidity Index may not be the most appropriate comorbidity index for patients in ICU, but was selected due to its focus on pre-existing chronic diseases as an indicator of complexity of morbidity. Fifth, various potential risk factors might have been more relevant in one outbreak than in another. A discriminative analysis on this matter, however, was not possible due to the limited number of patients per individual outbreak.

Conversely, we consider the stringent selection method for controls applied in the case-control study, and the fact that two controls per case were successfully identified for all except two cases, to be significant strengths of this study.

## Conclusions

Nosocomial acquisition of CRAB naturally is a multifactorial process. Nevertheless, this study demonstrated two principal risk factors for nosocomial CRAB acquisition in outbreak situations: sharing a patient room with a CRAB patient before availability of the microbiology report and undergoing bronchoscopy. Both findings could point to an underperformance of basic IPC measures, particularly regarding hand hygiene compliance and handling of medical devices. Our study reinforces the importance of these basic IPC measures and their continued promotion, particularly in outbreak situations. Given that the outbreaks occurred in the first year of the COVID-19 pandemic, our study serves as a reminder that stressful situations increase the likelihood of pathogen transmission, and that a focus on airborne precautions should not decrease the importance of other transmission-based precautions.

For 24 CRAB cases, two controls per case were successfully identified. For two CRAB cases, only one suitable control per case could be identified. Demographics and baseline characteristic of the 26 CRAB patients and 50 controls included in the case-control study are summarized in Table 2.

**Table 2:** Demographic and clinical characteristics of 26 patients with nosocomial carbapenem-resistant Acinetobacter baumannii (CRAB) acquisition and 50 matched controls

Parameter	Case (*N* = 26)	Control (*N* = 50)	p-value
	***N*(%)** or **Median (IQR)**	***N*(%)** or **Median (IQR)**	
Age in years	61 (54–68)	59 (50–67)	0.417
Male sex	21 (80.8%)	37 (74%)	0.510
Charlson Comorbidity Index	5 (3–7)	5 (4–7)	0.762
LOS on outbreak unit (days until discharge)	41 (29–57)	19 (8–44)	0.016
In-hospital death	13 (52%)	18 (36%)	0.138
Transfer from other hospital	17 (65.4%)	26 (52%)	0.264
Primary diagnosis: COVID-19	16 (61.5%)	26 (52%)	0.428
Observation period*	11 (5–23)	19 (8–44)	0.086
Invasive mechanical ventilation	25 (96.2%)	42 (84%)	0.120
Prone Position	17 (65.4%)	17 (34%)	0.009
ECMO during	12 (46.2%)	12 (24%)	0.049
Hemodialysis	12 (46.2%)	22 (44%)	0.858
Bronchoscopy	24 (92.3%)	33 (66%)	0.012
Sharing a patient room with a CRAB patient**	11 (42.3%)	2 (4%)	< 0.001
Contact to a CRAB room^#^	4 (15.4%)	0 (0%)	0.004
N of patients with CRAB on ICU until first CRAB detection or ICU discharge (median, IQR)	2 (1–6)	4 (2–8)	0.014
N of patients with CRAB on ICU per 10 days of stay (median, IQR)	0.18 (0.07–0.43)	0.2 (0.08–0.4)	0.827
Any antimicrobial treatment	24 (92%)	45 (90%)	0.741
Cephalosporine use	8 (32%)	25 (50%)	0.139
Fluorchinolone use	9 (34.6%)	16 (32%)	0.818
Carbapenem use	14 (53.8%)	27 (54%)	0.990

* Defined as the number of days on the outbreak ICU from ward admission until first CRAB detection (cases) or until ward discharge (controls)

** Defined as simultaneous admission to the same patient room before availability of the microbiological result

^#^ Defined as being admitted to a patient room previously occupied by a CRAB patient

Abbreviations: ECMO– extracorporeal membrane oxygenation, IQR - interquartile range, LOS– length of stay; N– number

The median observation period for cases was 11 days (IQR: 5–23) and for controls 19 days (IQR: 8–44). Descriptive analysis revealed that cases underwent certain invasive procedures (prone position, ECMO, bronchoscopy) significantly more frequently than controls. Concerning hemodialysis, no statistically significant difference was observed. CRAB cases were revealed to have shared a patient room with CRAB cases or to have been admitted to rooms where CRAB cases had been treated recently, significantly more often than controls. Contrarily, controls were exposed to a higher overall CRAB burden at the ward. Other parameters included in the descriptive analysis yielded no statistically significant differences between the groups.

Results of the univariable logistic regression analysis to determine whether observed differences represented a risk factor for nosocomial CRAB acquisition are displayed in Table 3. ECMO (OR: 5.1, *p* = 0.04), bronchoscopy (OR: 6.1, *p* = 0.02), and sharing a patient room with a CRAB patient before availability of the microbiological report (OR: 10.5, *p* < 0.01) were revealed to be factors significantly increasing the likelihood of nosocomial CRAB acquisition.

**Table 3:** Univariable analysis of risk factors for nosocomial carbapenem-resistant *Acinetobacter baumannii* (CRAB) acquisition

Parameter	Value	Odds Ratio	95% CI	p-value*
Age	Per year	1.02	0.98–1.06	0.318
Sex	Male	1.393	0.44–4.39	0.572
Transfer from other hospital	Yes	2.104	0.63–7.07	0.229
Primary diagnosis: COVID-19	Yes	2.241	0.56–8.95	0.253
Invasive mechanical ventilation	Yes	4.541	0.53–38.66	0.166
Prone position	Yes	nd	nd	0.995
ECMO	Yes	5.116	1.08–24.16	0.039
Hemodialysis	Yes	1.081	0.42–2.78	0.872
Bronchoscopy	Yes	6.145	1.3-28.96	0.022
Sharing a patient room with a CRAB patient*	Yes	10.543	2.33–47.67	0.002
Contact to a CRAB room**	Yes	nd	nd	0.993

* Defined as simultaneous admission to the same patient room before availability of the microbiological result

** Defined as being admitted to a patient room previously occupied by a CRAB patient

Abbreviations: CI– confidence interval; ECMO– extracorporeal membrane oxygenation; nd– not defined

Results of the multivariable analysis are summarized in Table 4. Two parameters were identified to be independent risk factors for nosocomial CRAB acquisition. Sharing a patient room with a CRAB patient before availability of the microbiological report was associated with a more than tenfold increase in the risk of nosocomial CRAB acquisition (OR: 10.7, CI: 2.3–50.9, *p* = 0.01), while undergoing bronchoscopy increased the risk more than six times (OR: 6.9, CI: 1.3–38.1, *p* < 0.01).

**Table 4:** Multivariable analysis of risk factors for nosocomial carbapenem-resistant *Acinetobacter baumannii* acquisition

Parameter	Odds Ratio	95% CI	p-value
Bronchoscopy	6.922	1.26–38.07	< 0.001
Sharing a patient room with a CRAB patient*	10.698	2.25–50.89	0.014

* Defined as simultaneous admission to the same patient room

Abbreviations: CI– confidence interval

## Data Availability

No datasets were generated or analysed during the current study.

## Abbreviations

CI: Confidence interval(s)
CRAB: Carbapenem-resistant *Acinetobacter baumannii*
ECMO: Extracorporeal membrane oxygenation
ICU: Intensive care unit(s)
IPC: Infection prevention and control
IQR: Interquartile range(s)
MDRO: Multidrug-resistant organism(s)
OR: Odds ratio(s)
